# Qingfei Dayuan granules and decoction alleviate acute lung injury via TLR4 signaling pathway modulation, gut microbiota regulation, and metabolic reprogramming

**DOI:** 10.3389/fphar.2025.1643544

**Published:** 2025-11-12

**Authors:** Huanbo Cheng, Jingwen Ha, Yuanming Ba

**Affiliations:** 1 Affiliated Hospital of Hubei University of Chinese Medicine, Hubei Provincial Hospital of Traditional Chinese Medicine, Wuhan, Hubei, China; 2 Hubei Shizhen Laboratory, Wuhan, Hubei, China; 3 Hubei Key Lab of Quality and Safety of Traditional Chinese Medicine and Health Food, Huangshi, China; 4 School of Traditional Chinese Pharmacy, China Pharmaceutical University, Nanjing, China

**Keywords:** Qingfei Dayuan granules, Qingfei Dayuan decoctions, acute lung injury, gut microbiota, TLR4, metabolomics

## Abstract

**Objective:**

To explore and compare the therapeutic effects and underlying mechanisms of Qingfei Dayuan Granules (QFDYGs) and Qingfei Dayuan Decoctions (QFDYDs) for the treatment of acute lung injury (ALI), focusing on the modulation of the TLR4 signaling pathway, intestinal microbiota, and related metabolic pathways.

**Methods:**

The active metabolite contents were measured by ultrahigh-performance liquid chromatography. A mouse model of lipopolysaccharide-induced ALI (tracheal instillation) was used to assess efficacy. After drug administration, lung tissue damage was analyzed by hematoxylin-eosin staining and quantification of inflammatory cytokines and oxidative stress biomarkers with enzyme-linked immunosorbent assays. Components of the TLR4 signaling pathway were quantified by Western blot analysis. Intestinal flora regulation was assessed by 16S rRNA sequencing with metabolic pathway analysis via metabolomics. Multivariate statistical methods were applied to analyze differences in gut microbiota and metabolites between groups.

**Results:**

Levels of eight metabolites were 2.44–3.74 times greater following treatment with QFDYGs vs. QFDYDs, although both demonstrated significant protective effects against pulmonary inflammation through TLR4 signaling pathway modulation, gut microbiota restoration, and metabolic regulation. QFDYDs more effectively suppressed production of the pro-inflammatory cytokines TNF-α and IL-1β, while QFDYGs exhibited superior capability to reduce malondialdehyde levels and restore glutathione, catalase, and superoxide dismutase activities. QFDYGs demonstrated greater inhibition of the TLR4-TRIF/MyD88-NF-κB-NLRP3 signaling pathway, whereas QFDYDs more effectively normalized lung injury-induced metabolic changes. Both formulations significantly modulated metabolic pathways, as evidenced by sustained changes to 11 key metabolites, and improved intestinal microbiota composition and functionality.

**Conclusion:**

Both QFDYGs and QFDYDs offer protection against ALI. QFDYGs could serve as effective alternatives to QFDYDs, with equivalent or potentially superior therapeutic effects. The choice between QFDYGs and QFDYDs should be guided by specific clinical presentations and therapeutic goals.

## 1 Introduction

Acute lung injury (ALI) is a critical condition characterized by severe onset of hypoxic respiratory insufficiency, stemming from damage to alveolar epithelial cells and pulmonary capillary endothelial cells due to a variety of direct and indirect injurious factors, resulting in diffuse interstitial and alveolar edema, severely compromising respiratory function (Fanelli and Ranieri, 2015; Fang et al., 2025). ALI often progresses to acute respiratory distress syndrome, a more severe form that was first described in 1967 as a complex cascade of pathological processes, and carries a high 28-day mortality rate of approximately 20%–40% (Yehya et al., 2023). ALI can be triggered by infection, collagen vascular disease, drug-induced injury, toxin ingestion, inhalational exposure, shock, acute eosinophilic pneumonia, immune-mediated pulmonary hemorrhage and vasculitis, as well as radiation pneumonitis (Long et al., 2022). Currently, corticosteroids with effective anti-inflammatory properties, such as dexamethasone (DEX) and prednisone, are clinically administered to manage ALI. However, those drugs are often accompanied by a range of adverse effects and serious reactions, thereby limiting therapeutic utility (Lv et al., 2023). Despite advancements in supportive care, especially improved ventilator management and critical care strategies, the morbidity and mortality rates of ALI remain unacceptably high (Wu et al., 2024). The diverse etiology and multifactorial pathogenesis of ALI pose significant challenges to the development of effective treatment strategies, highlighting the urgent need for innovative pharmacological interventions to improve clinical outcomes and reduce the global burden of ALI (Zhao and Bie, 2022). Consequently, there is a pressing demand for intensified research efforts to discover new agents to target the intricate mechanisms underlying the progression of ALI.

As compared to modern medicine, traditional Chinese medicine (TCM) can offer a protective effect against ALI by acting on multiple targets and pathways (Lv et al., 2023; Shi et al., 2023). In TCM theory, “heat, toxicity, phlegm, and stasis” are components of the development and progression of ALI, whereas “phlegm and stasis” constitute the pathological basis (Ding et al., 2020). Qingfei Dayuan Granules (QFDYGs), formerly known as “Pneumonia Formula No. 1”, produced by State Physician Prof. Guoqiang Mei under guidance of the Chinese medicine epidemic theory, are based on the classic Xiaochaihu and Dayuanyin decoctions with modifications to reduce phlegm to clear the lungs and alleviate asthma (Huanbo et al., 2023). Clinical studies have demonstrated that QFDYGs can rapidly reduce fever, improve lung imaging signs, and reverse lung inflammation (Li et al., 2023; Zhu et al., 2024). Despite clinical efficacy, there is a notable lack of systematic research on the mechanisms underlying the beneficial effects of QFDYGs for treatment of ALI, especially comprehensive analysis of potential molecular targets, biological processes, and metabolic pathways.

Qingfei Dayuan Decoctions (QFDYDs), the classical decoctions, conserve the full water-extract chemical profile and are often considered to have a rapid onset of action due to the direct availability of bioactive metabolites. Yet they retain excessive amounts of high-molecular-weight polysaccharides and peptides, resulting in variable inter-individual bioavailability and noticeable batch-to-batch inconsistency (Cai et al., 2024). In contrast, the modernized QFDYGs introduce additional purification and spray-drying steps that remove most polysaccharides and proteins while enriching a medium-polarity fraction of flavonoid–saponin clusters such as baicalin and timosaponin BII (Cheng et al., 2024). QFDYGs offer accurate dosing, lower dosage volume, easy portability and rapid dissolution. Although clinicians often use the two forms interchangeably, experimental evidence of equivalent efficacy and guidance on when to prefer one over the other is still lacking. This study is the first to conduct a side-by-side comparison of both formulations in an ALI model. A comparative evaluation of the efficacy and mechanisms of the granule versus decoction forms could provide valuable insights to optimize TCM-based therapies against ALI.

In the present study, the protective effects of QFDYGs and QFDYDs against ALI were systematically explored using a mouse model of lipopolysaccharide (LPS)-induced ALI and modern analytical techniques. Clarification of the impact of QFDYGs and QFDYDs on the lung histopathology and inflammatory factors of the experimental mice provides important preliminary insights into the underlying protective mechanisms, offering a theoretical foundation for further exploration of potential clinical applications.

## 2 Materials and methods

### 2.1 Reagents and standards

Enzyme-linked immunosorbent assay (ELISA) kits for quantification of mouse tumor necrosis factor-α (TNF-α), interleukin 6 (IL-6), and interleukin 1β (IL-1β), as well as the cellular contents of malondialdehyde (MDA), myeloperoxidase (MPO), glutathione (GSH), catalase (CAT), and superoxide dismutase (SOD) were sourced from Jiangsu Meimian Industrial Co., Ltd., (Jiangsu, China). Antibodies against toll-like-receptor 4 (TLR4), molecule myeloid differentiation factor 88 (MyD88), tir-domain-containing adaptor inducing interferon-B (TRIF), nuclear factor-κB (NF-Κb) p65, phosphorylated (p)-NF-κB p65, and the nucleotide binding and oligomerization domain-like receptor family pyrin domain-containing 3 (NLRP3) were procured from Hunan Aifang Biotech Co., Ltd., (Changsha, China). Ultrahigh-performance liquid chromatography (UHPLC)-grade acetonitrile, methanol, and formic acid were obtained from Merck KGaA (Darmstadt, Germany). L-2-chlorophenylalanine (internal standard) was obtained from Shanghai Yuanye Bio-Technology Co., Ltd., (Shanghai, China). All other reagents were of analytical grade. Reference substances (procyanidin B1, mangiferin, paeoniflorin, polydatin, hesperidin, baicalin, timosaponin BII, and baicalein) were acquired from the China National Institute for Food and Drug Control (Beijing, China).

### 2.2 Preparation of QFDYGs

The QFDYGs were composed of 13 medicinal herbs: *Bupleurum chinense* DC. [Apiaceae; Bupleuri Radix]; *Scutellaria baicalensis* Georgi [Lamiaceae; Scutellariae radix]; *Amomum tsao-ko* Crevost et Lemaire [ Zingiberaceae; Tsaoko Fructus]; *Magnolia officinalis* Rehd. et Wils. [Magnoliaceae; Magnoliae Officinalis Cortex]; *Areca catechu* L. [Arecaceae; Arecae semen]; *Citrus reticulata* Blanco [Rutaceae; Citri Reticulatae Pericarpium]; *Pinellia ternata* (Thunb.) Breit. [Araceae; Pinelliae Rhizoma Praeparatum]; *Paeonia lactiflora* Pall. [Paeoniaceae; Paeoniae radix rubra]; *Anemarrhena asphodeloides* Bge. [Asparagaceae; Anemarrhenae Rhizoma]; *Codonopsis pilosula* (Franch.) Nannf. [Campanulaceae; Codonopsis Radix]; *Trichosanthes kirilowii* Maxim. [Cucurbitaceae; Trichosanthis Fructus]; *Polygonum cuspidatum* Siebold and Zucc. [Polygonaceae; Polygoni Cuspidati Rhizoma et Radix]; and *Glycyrrhiza uralensis* Fisch. [Leguminosae; Glycyrrhizae Radix et Rhizoma]. All herbs were taxonomically identified by Prof. Keli Chen (Hubei University of Chinese Medicine, Wuhan, China). The QFDYDs were prepared by water extraction and vacuum concentration. As “Type A extracts” (Li et al., 2023), after concentration, the QFDYGs were further extracted three times with saturated *n*-butanol, purified via ceramic membrane filtration, followed by spray drying and dry-granulation. Then, 25 g of the medicinal herbs were produced into 1 g of QFDYGs or 2.9 g of QFDYDs. All samples were supplied by Jing Brand Chizhengtang Pharmaceutical Co., Ltd., (Daye, China).

### 2.3 UHPLC analysis of active metabolites in QFDYGs and QFDYDs

The samples (0.05 g) were dissolved in 5 mL of 50% ethanol, sonicated for 20 min, cooled to room temperature, shaken thoroughly, filtered through a 0.22-μm syringe filter, and then injected into a Waters ACQUITY UHPLC H-Class system (Waters Corporation, Milford, MA, USA). The chromatographic conditions were based on existing research, with minor modifications (Cheng et al., 2024). A series of solutions at various concentrations of the reference substances procyanidin B1 (2.25–112.54 µg/mL), mangiferin (3.06–150.54 µg/mL), paeoniflorin (12.57–628.50 µg/mL), polydatin (1.60–80.16 µg/mL), hesperidin (8.72–436.14 µg/mL), baicalin (75.26–1505.22 µg/mL), timosaponin BII (4.48–224.07 µg/mL), and baicalein (10.21–306.33 µg/mL) were prepared to construct standard curves. All solutions were stored at 4 °C prior to UHPLC analysis.

### 2.4 Animals group and treatment

Specific-pathogen-free (SPF) male Balb/c mice (n = 70; body weight, 20–22 g) were supplied by Jiangsu Huachuang Xinnuo Pharmaceutical Science and Technology Co., Ltd. (Jiangsu, China; certificate no. SCXK (Su) 2020-0009) and housed at 10 per cage in an SPF laboratory at China Pharmaceutical University under a constant temperature of 22 °C ± 2 °C and humidity of 50% ± 15% with *ad libitum* access to standard rodent chow and water. The study protocol was approved by the Institutional Animal Care and Use Committee of China Pharmaceutical University (approval no. 2022-08-026) and conducted in accordance with the “Guide for the Care and Use of Laboratory Animals”.

The mice were randomly assigned to one of seven groups (n = 10): control, model, positive control (DEX), high-dose QFDYGs (h-QFDYGs), low-dose QFDYGs (l-QFDYGs), high-dose QFDYDs (h-QFDYDs), and low-dose QFDYDs (l-QFDYDs). During the 3-day experiment, the mice were treated with DEX (9.75 mg/kg), h-QFDYGs (0.34 g/kg), h-QFDYDs (0.90 g/kg), l-QFDYGs (0.17 g/kg), or l-QFDYDs (0.45 g/kg) as protective therapy, respectively, while the control and model groups were orally administered equal volumes of 0.9% NaCl. On day 2, all groups, except the control group, were subjected to LPS-induced airway infusion (2 mg/g) as previously described (Tan et al., 2024). On day 3, after the final administration, the mice were anesthetized and blood was collected from the eyeballs. The drug-containing serum was obtained via centrifugation at 2,500 rpm for 15 min. Alveolar lavage fluid, lung tissues, and cecum contents were collected. The lung tissues were fixed in 4% paraformaldehyde. The wet/dry (W/D) weight ratio of the right lower lung was calculated to assess pulmonary edema.

### 2.5 Pathological examination

Lung tissues were removed from the fixative solution, rinsed thoroughly, dehydrated, embedded in paraffin, and cut into sections, which were stained with hematoxylin and eosin (H&E), mounted on slides, and examined under a light microscope. A blinded semiquantitative lung injury score based on the 2011 American Thoracic Society (ATS) consensus was performed (Matute-Bello et al., 2011).

### 2.6 Detection of inflammatory cytokines and oxidative stress biomarkers

TNF-α, IL-6, IL-1β, and MPO levels in bronchoalveolar lavage fluid (BALF), as well as MDA and GSH levels in serum and CAT and SOD activities were detected using ELISA kits per the manufacturer’s instructions.

### 2.7 Western blotting analysis

The quantity of proteins extracted from cells and lung tissues was determined with a bicinchoninic acid protein assay kit. Equal amounts of protein (40 μg) were separated by electrophoresis with 10%–15% sodium dodecyl sulfate gels and subsequently electroblotted onto polyvinylidene fluoride membranes, which were blocked with 5% bovine serum albumin, and incubated overnight at 4 °C with primary antibodies, followed by incubation with horseradish peroxidase-conjugated secondary antibodies. The protein bands were then visualized and analyzed using Image Lab™ Software (Bio-Rad Laboratories, Hercules, CA, USA).

### 2.8 16S rRNA sequencing assay

A library of 16S rRNA gene sequences was constructed by Biomarker Biotechnology Co., Ltd., (Beijing, China). Specific barcoded primers were designed based on the full-length primer sequences. Products of PCR amplification were purified, quantified, and normalized. The library was quality checked prior to sequencing with the PacBio Sequel system (Pacific Biosciences, Menlo Park, CA, USA). The data were preprocessed for subsequent bioinformatics analysis.

### 2.9 Metabolomics assay

Drug-containing serum was mixed with a methanol solution containing 3% L-2-chlorophenylalanine, vortexed for 1 min, incubated on ice for 20 min, and centrifuged at 14,000 rpm for 15 min. Metabolomics analysis was performed on a Xevo G2-XS QTOF system (Waters Corporation) with the same mass spectrometry settings as in our previous studies. Mass spectrometry data were subjected to peak alignment to eliminate unstable components, thereby acquiring the retention time, *m/z* values, and ion peak intensity of each metabolite. Subsequently, differential metabolites were identified through chemometric analysis. Finally, metabolic pathway analysis of the differential metabolites was performed using MetaboAnalyst 5.0 software (https://www.metaboanalyst.ca).

### 2.10 Statistical analysis

One-way analysis of variance, box plot generation, and Pearson’s correlation analysis were performed using OriginPro 2024b software (OriginLab Corporation, Northampton, MA, USA). Principal component analysis (PCA), orthogonal partial-least-squares discriminant analysis (OPLS-DA), and permutation testing were performed, and the variable and importance in the projection (VIP) scores were examined using SIMCA 14.1 software (Umetrics AB, Umea, Sweden).

## 3 Results

### 3.1 Quantitative determination results of active metabolites in QFDYGs and QFDYDs

**FIGURE 1:**
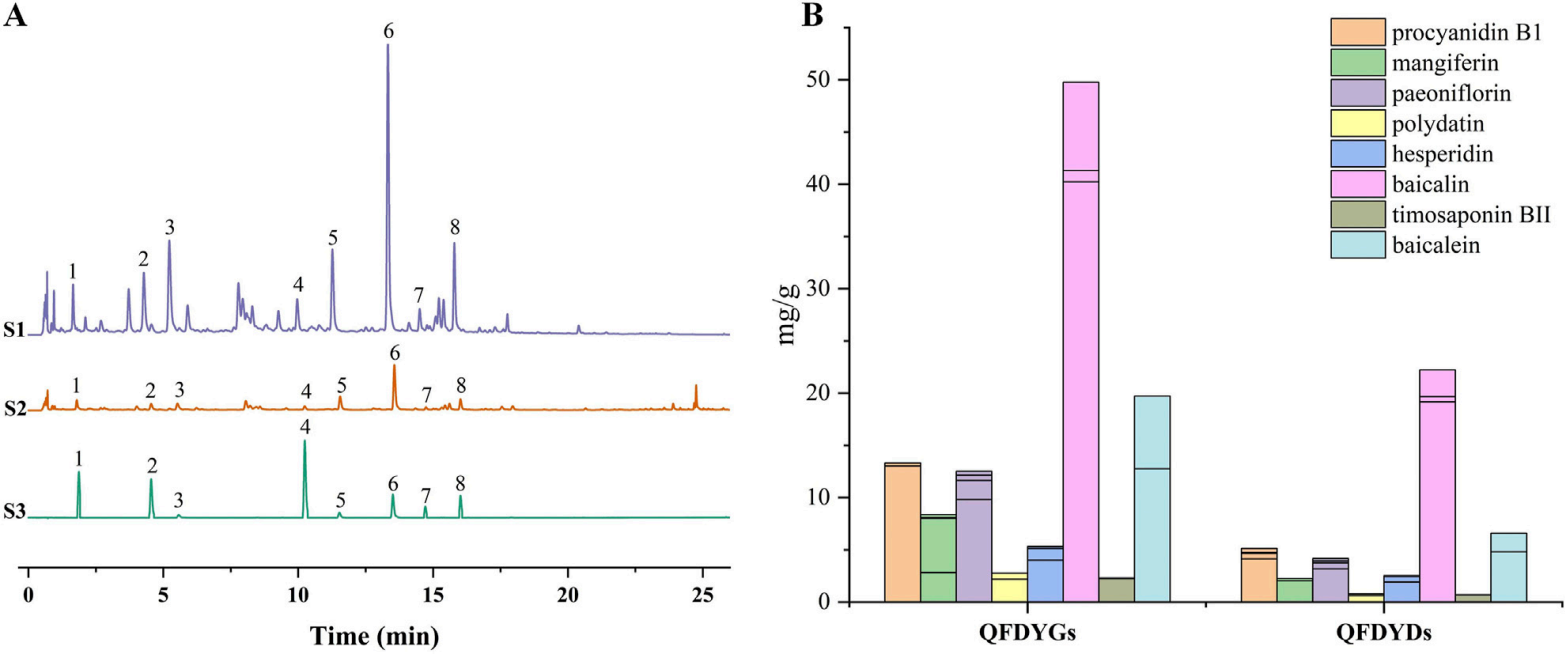
Quantification of active metabolites. **(A)** UHPLC chromatograms of QFDYGs, QFDYDs, and references substances. Peaks 1 to 8 represent procyanidin B1, mangiferin, paeoniflorin, polydatin, hesperidin, baicalin, timosaponin BII, and baicalein, respectively. **(B)** Contents of the eight metabolites. Data are presented as mean ± SD, n = 6.

Using UHPLC-Q-TOF-MS, we previously identified 91 metabolites in the prescription (Cheng et al., 2024). In this study, eight major active metabolites, including procyanidin B1, mangiferin, paeoniflorin, polydatin, hesperidin, baicalin, timosaponin BII, and baicalein, were quantified. Quantitative analysis demonstrated *R* ^2^ > 0.9990 for all calibration curves and the eight active metabolites had a separation greater than 1.5. The relative standard deviation values for precision, repeatability, and stability of the experiments, and the recovery ranges met the requirements of methodological research, demonstrating that the analytical method is reliable and reproducible. Chromatograms of QFDYGs, QFDYDs, and reference substances are provided in Figure 1A. After purification, the contents of the eight metabolites were 2.44–3.74-fold greater in QFDYGs than QFDYDs (Figure 1B).

### 3.2 Effects of QFDYGs and QFDYDs on lung histopathological damage

**FIGURE 2:**
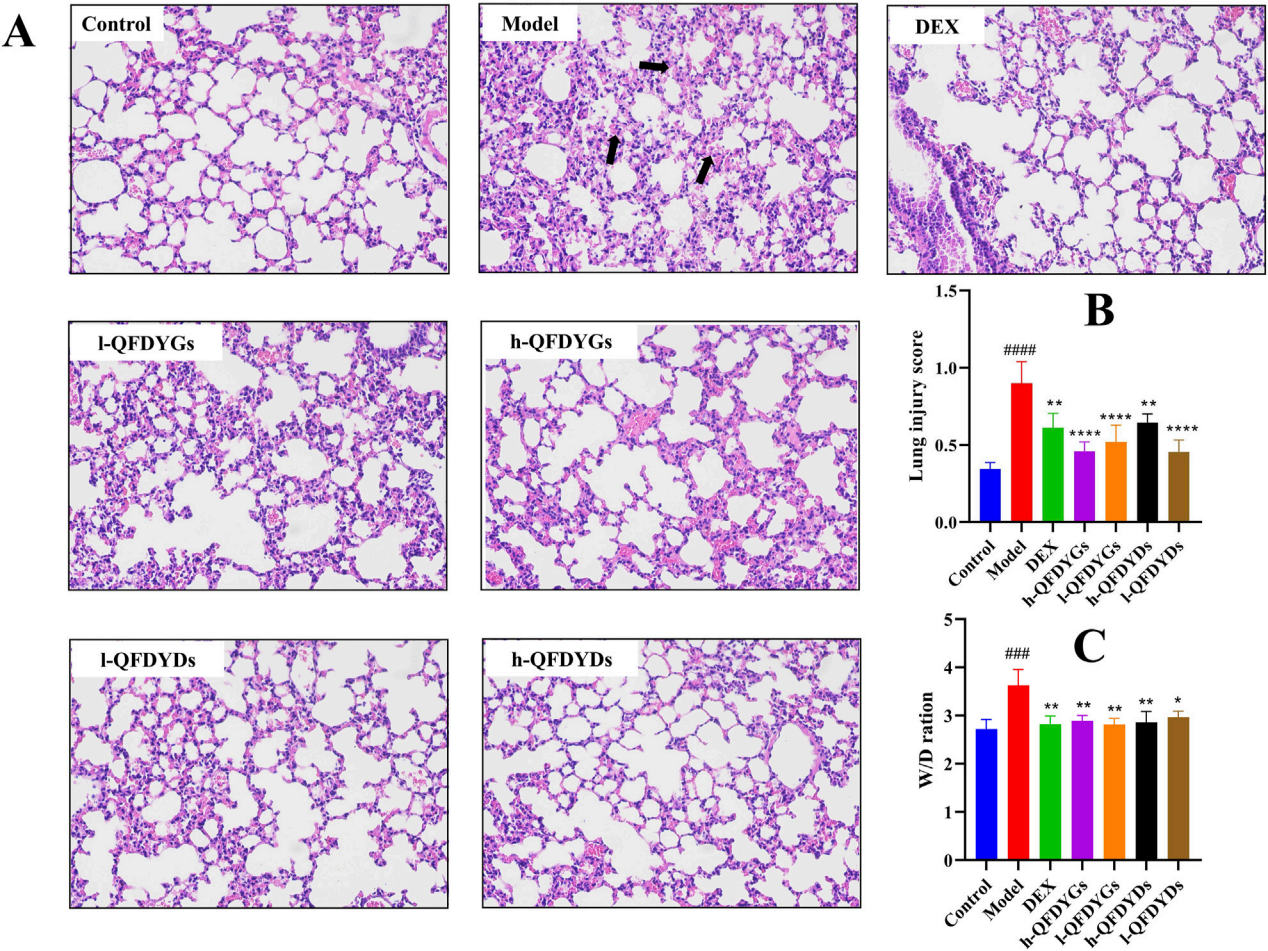
QFDYGs and QFDYDs alleviated lung pathological damage and lowered the W/D ratio. **(A)** Representative histologic H&E-stained lung sections (×200). The black arrow highlights severe alveolar structural damage, alveolar cavity hemorrhage, and inflammatory cell infiltration. **(B)** Lung injury score. **(C)** W/D ratios. Data are presented as mean ± SD, n = 6. ^*###*^ *p* < 0.001 vs. control group; *p < 0.05, **p < 0.01, ****p < 0.0001 vs. model group.

Histopathological assessment via H&E staining showed the model group had severe alveolar structural damage, with alveolar hemorrhage, inflammatory cell infiltration, interstitial edema, and much thicker alveolar septa than the control group (Figure 2A). Figure 2B shows the mean lung injury scores for histopathological evaluation of the lung tissues in the groups. Treatment with QFDYGs and QFDYDs greatly lessened LPS-induced lung morphological damage with significant difference between groups. As compared to the control group, the W/D ratio was significantly elevated in the model group, indicative of pulmonary edema (*p* > 0.001). Yet, treatment with QFDYGs and QFDYDs markedly lowered the W/D ratio (*p* < 0.05) versus the model group, indicating reduced pulmonary edema (Figure 2C). Also, within the tested doses, treatment with QFDYGs and QFDYDs did not produce discernible changes in body weight, food intake, fur quality or other behavioral abnormalities, suggesting an absence of overt behavioral or systemic adverse effects under our experimental settings. Overall, both formulations alleviated LPS-induced ALI, with similar histopathological and edema improvement.

### 3.3 Regulation of pulmonary inflammation and oxidative stress by QFDYGs and QFDYDs

**FIGURE 3:**
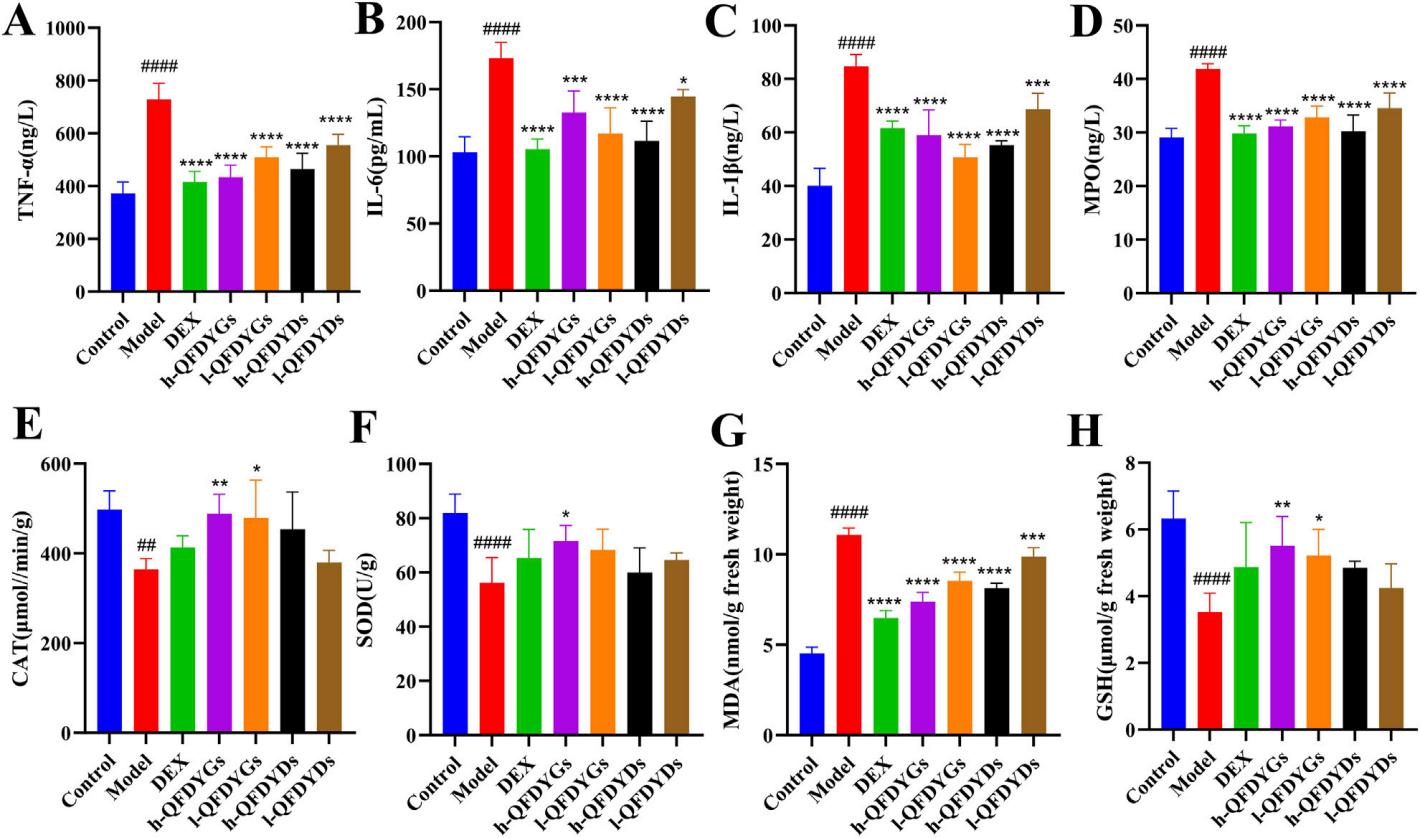
Levels of inflammatory cytokines in BALF and oxidative stress biomarkers in serum. **(A)** TNF-α, **(B)** IL-6, **(C)** IL-1β, **(D)** MPO, **(E)** CAT, **(F)** SOD, **(G)** MDA, and **(H)** GSH. Data are presented as mean ± SD, n = 6. ^*##*^ *p* < 0.01, ^*####*^ *p* < 0.0001 vs. control group; *p < 0.05, **p < 0.01, ***p < 0.001, ****p < 0.0001 vs. model group.

Cytokine levels in BALF were measured to assess how QFDYGs regulate inflammation and immune responses in mice with LPS-induced ALI. As compared to the control group, the model group had significantly higher levels of TNF-α, IL-6, IL-1β, and MPO (Figures 3A–D), confirming successful generation of a mouse model of LPS-induced ALI. Treatment with QFDYGs and QFDYDs markedly reduced production of these inflammatory factors as compared to the model group. Of note, as compared to h-QFDYGs, h-QFDYDs more effectively suppressed production of IL-6, IL-1β, and MPO. Also, both formulations showed dose-dependent inhibition of TNF-α secretion.

Oxidative stress biomarkers were assessed to further explore the antioxidant potential of QFDYGs against ALI. The model group had higher MDA levels (*p* < 0.0001) and lower activities of CAT, SOD, and GSH (Figures 3E–H). DEX and both formulations of QFDYGs reversed these changes. Specifically, QFDYGs more effectively reduced MDA and restored CAT, SOD, and GSH activities than QFDYDs, but to varying degrees.

### 3.4 QFDYGs and QFDYDs modulated the TLR4 signaling pathway

**FIGURE 4:**
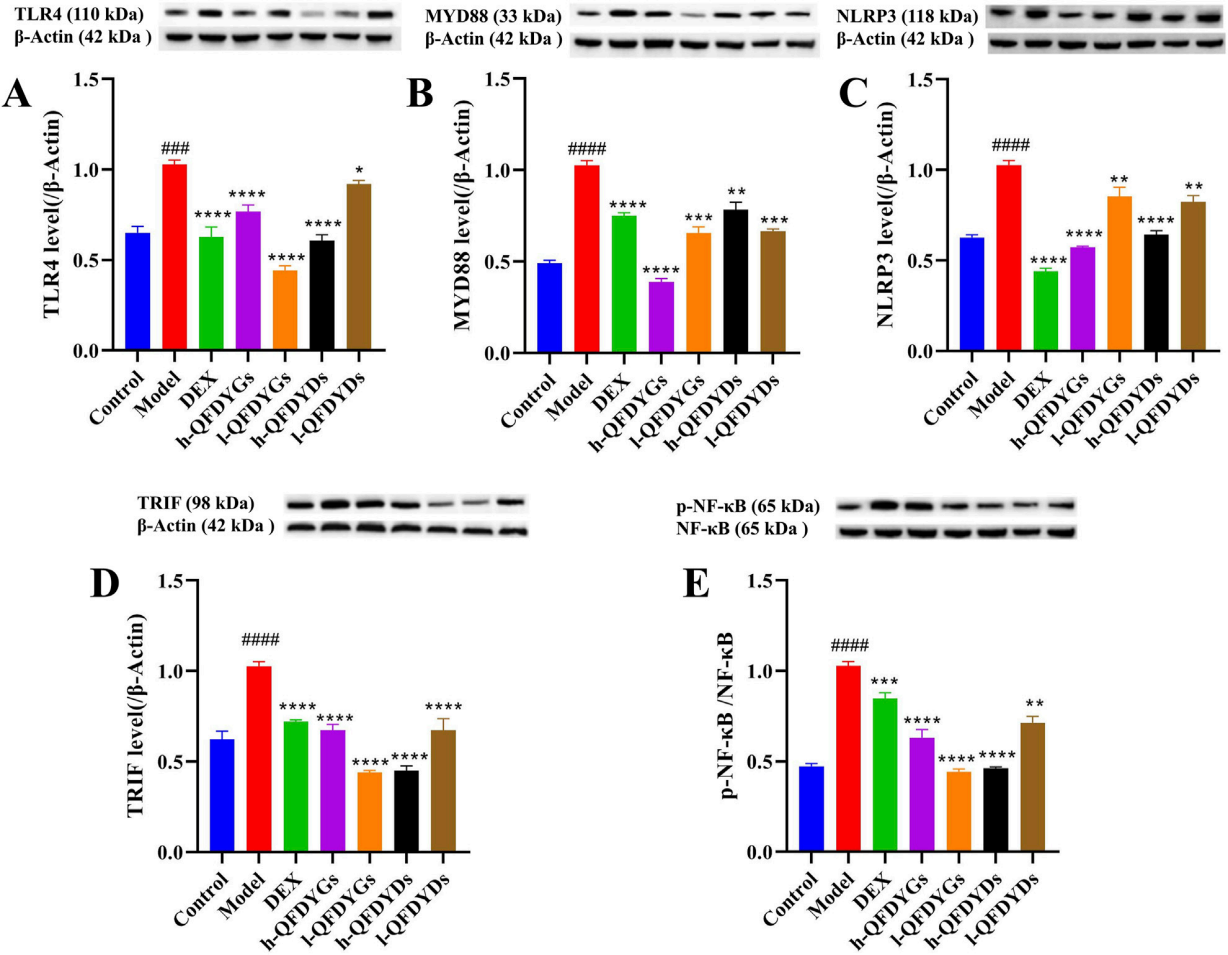
Western blot analysis of the components of the TLR4 signaling pathway following treatment with QFDYGs and QFDYDs. **(A)** TLR4, **(B)** MyD88, **(C)** NLRP3, **(D)** TRIF, and **(E)** p-NF-κB/NF-κB. Data are presented as mean ± SD, n = 3. ^*###*^ *p* < 0.001, ^*####*^ *p* < 0.0001 vs. control group; *p < 0.05, **p < 0.01, ***p < 0.001, ****p < 0.0001 vs. model group.

Relative to the control group, the model group demonstrated considerably higher expression of TLR4, MyD88, TRIF, NLRP3, and p-NF-κB (Figures 4A–E), indicating successful activation of the TLR4-TRIF/MyD88 NF-κB-NLRP3 signaling pathway in LPS-induced ALI. As compared to the model group, treatment with both QFDYGs and QFDYDs markedly downregulated these proteins and suppressed NF-κB phosphorylation. The effects of QFDYGs and QFDYDs on activation of the TLR4-TRIF/MyD88 NF-κB-NLRP3 signaling pathway were examined by Western blot analysis. Notably, h-QFDYGs more effectively suppressed MyD88 expression than h-QFDYDs, while h-QFDYDs better inhibited TRIF expression. Moreover, QFDYGs showed better performance than QFDYDs in suppressing NF-κB phosphorylation. QFDYDs exhibited a relatively clear dose-dependent effect (except for MYD88), whereas QFDYGs did not. In fact, for TLR4, TRIF and p-NF-κB, l-QFDYG consistently showed stronger inhibition than h-QFDYG. Overall, these results suggest that QFDYGs and QFDYDs modulate key components of the TLR4-TRIF/MyD88-NF-κB-NLRP3 pathway to different extents.

### 3.5 Impact of QFDYGs and QFDYDs on gut microbiota composition and abundance

**FIGURE 5:**
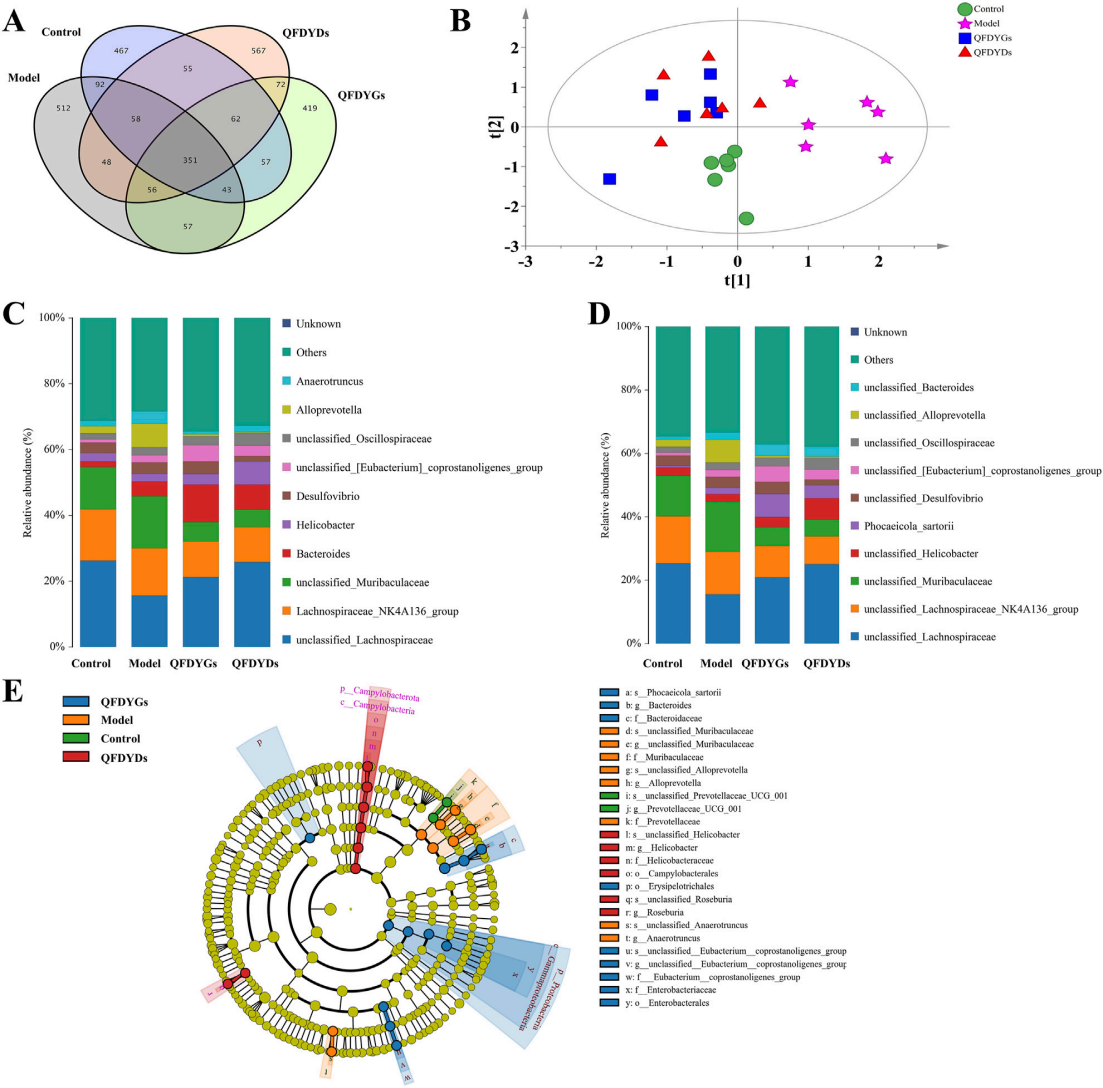
Diversity analysis of the gut microbiota. **(A)** Venn diagram showing different strains identified by OTUs analysis. **(B)** PCA of beta diversity analysis. **(C,D)** Relative abundances and composition of microbial communities at the genus and species levels. **(E)** Linear Discriminant Analysis of Effect Size analysis. Data are presented as mean ± SD, n = 6.

Representative images of gels of separated DNA of the samples are provided in Supplementary Figure S1. Dilution curves and sequencing abundance curves were generated to evaluate the effects of QFDYGs and QFDYDs on the richness, evenness, homogeneity, and diversity of gut microbial communities in ALI mice (Supplementary Figure S2). The sequencing depth achieved in this study was sufficient to cover nearly all species within the samples (Liu et al., 2023). In total, 351 operational taxonomic units (OTUs) were common across the four groups based on a 97% similarity threshold, highlighting overlapping of the microbial communities (Figure 5A). The QFDYGs and control groups shared more OTUs than the QFDYGs and model groups. Based on the OTUs, the Chao1 and Shannon diversity indices were significantly lower in the model group than the control group. In contrast, treatment with both QFDYGs and QFDYDs restored these indices to levels closer to those of the control group (Supplementary Table S1). PCA further confirmed distinct clustering of microbiota among the groups (Figure 5B). Both QFDYGs and QFDYDs increased the abundance and diversity of the gut flora, effectively ameliorating intestinal microbiota dysbiosis induced by ALI. Notably, no significant differences were observed between QFDYGs and QFDYDs in terms of the restorative effects on gut microbiota composition or diversity.

The impact of QFDYGs on gut microbiota composition was assessed at the phylum, genus, and species levels, focusing on taxa with higher abundances and proportional distributions. At the phylum level, the dominant microbiomes identified were Bacteroidetes, Firmicutes, and Campylobacterota (Supplementary Figure S3A). At the genus and species levels (Figures 5C,D), the predominant taxa included *unclassified_Lachnospiraceae*, *Lachnospiraceae*_NK4A136_group, and *unclassified_Muribaculaceae*. As compared to the control group, the model group exhibited significant downregulation in the relative abundances of *Firmicutes* and *unclassified_Lachnospiraceae*, alongside upregulation of the proportions of *Bacteroidetes*, *unclassified_Muribaculaceae*, and *Alloprevotalla* (Supplementary Figure S3B). Treatment with QFDYGs and QFDYDs significantly reversed these alterations, though with notable differences. Specifically, QFDYGs demonstrated superior efficacy in restoring the abundance of *Firmicutes*, while QFDYDs more effectively upregulated the proportion of *unclassified_Muribaculaceae* and downregulated the abundances of *Bacteroidetes*, *Alloprevotalla*, and *unclassified_Lachnospiraceae*. Both QFDYGs and QFDYDs ameliorated microbiota dysbiosis to varying degrees, as the distribution of the gut flora was closer to that of the healthy control group. The results of Linear Discriminant Analysis of Effect Size were basically consistent. *Alloprevotalla*, *Prevotellaceae*, *unclassified_Anaerotruncus*, and *unclassified_Muribaculaceae* were significantly overrepresented in the intestinal flora of mice in the model group as compared to the control group. After administration of QFDYGs, the proportions of all intestinal flora decreased to varying degrees (Figure 5E). These findings highlight the differential effects of QFDYGs and QFDYDs on specific microbial taxa, suggesting that the mechanisms of action may involve distinct pathways involved in microbiota modulation.

The ALI-induced contraction of Firmicutes and expansion of Bacteroidetes observed here mirror the dysbiosis signature repeatedly associated with critical illness and poor pulmonary outcome. Firmicutes are the principal source of short-chain fatty acids such as butyrate and propionate, these metabolites reinforce epithelial tight-junction proteins, dampen TLR4-TRIF/MyD88-NF-κB-NLRP3 signalling, and promote the differentiation of anti-inflammatory Treg cells (Yin et al., 2025; Yu et al., 2025). Their relative depletion therefore weakens both intestinal and alveolar barrier integrity, facilitating systemic endotoxin translocation that further amplifies lung inflammation. Conversely, the bloom of Bacteroidetes, especially the genus *Alloprevotella*, is not merely a by-stander effect. *Alloprevotella* plays the role of a “combustion aid” in the inflammatory chain of ALI (Yang et al., 2025). Animal experiments had shown that for every 1 log increase in *Alloprevotella*, serum LPS rises by approximately 0.3 EU/mL, which is positively correlated with IL-6 and TNF-α in BALF (Zhu et al., 2025). Within Firmicutes we recorded a selective loss of *unclassified_Lachnospiraceae* and *Blautia*, two clades whose abundance inversely correlates with IL-6/IL-1β concentration in murine pneumonia models (Yin et al., 2025). Restoration of these taxa by QFDYGs therefore provides a mechanistic explanation for the parallel reduction in lung W/D ratio and pro-inflammatory cytokines (Figures 2, 3). The observed decrease in these taxa after QFDYGs treatment aligns with the downregulation of pulmonary NLRP3 and p-NF-κB (Figure 4) and provides microbiome-based evidence for the formulations’ protective efficacy.

### 3.6 Influence of QFDYGs and QFDYDs on metabolic profiles

**FIGURE 6:**
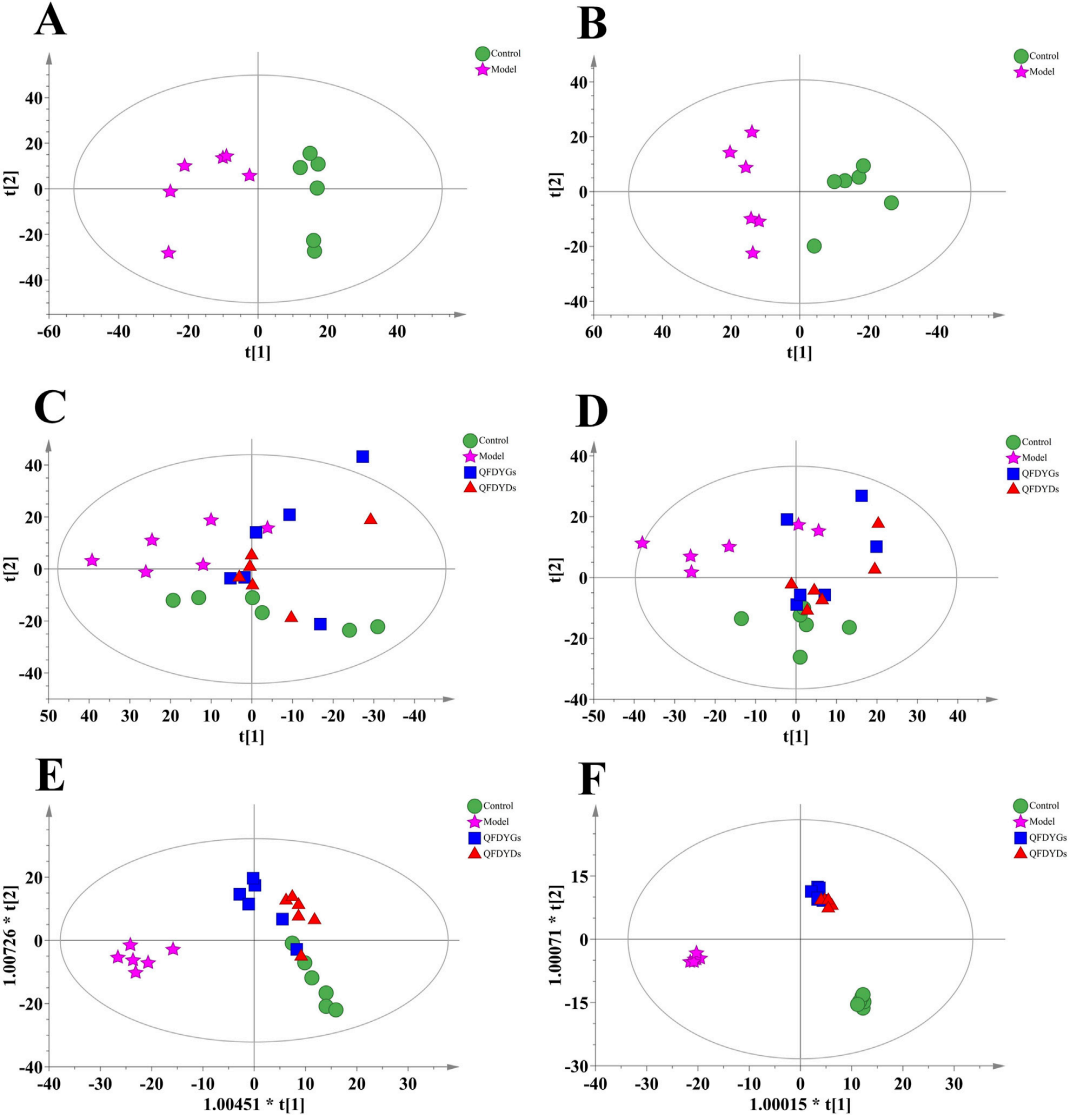
Metabolic profiles based on PCA and OPLS-DA. **(A)** PCA score plots in positive ion mode of control-model groups. **(B)** PCA score plots in negative ion mode of control-model groups. **(C)** PCA score plots in positive ion mode of control-model-QFDYGs-QFDYDs groups. **(D)** PCA score plots in negative ion mode of control-model-QFDYGs-QFDYDs groups. **(E)** OPLS-DA score plots in positive ion mode of control-model-QFDYGs-QFDYDs groups. **(F)** OPLS-DA score plots in negative ion mode of control-model-QFDYGs-QFDYDs groups. Data are presented as mean ± SD, n = 6.

Metabonomics is a reliable tool to assess changes to multiple targets, components, and pathways influenced by TCM (Li et al., 2022; Wang et al., 2017). PCA of serum metabolic information in positive/negative ion modes separated the control and model groups (Figures 6A,B), indicating severely disrupted normal physiological function in mice after lung injury. However, there was no distinct separation of the four groups (Figures 6C,D). OPLS-DA, a supervised modeling method, showed good recognition performance. In positive ion (*R* ^2^X = 0.555, *R* ^2^Y = 0.815, *Q* ^2^ = 0.721) and negative ion (*R* ^2^X = 0.662, *R* ^2^Y = 0.711, *Q* ^2^ = 0.282) modes, the control, model, and treatment groups were distinctly separated. As shown in Figures 6E,F, under both ion modes, model group samples clustered to the left and the control group to the right. The groups treated with QFDYGs and QFDYDs leaned towards the control group. As compared to the groups treated with QFDYGs, those treated with QFDYDs were closer to the control group and farther from the model group. This shows that both formulations caused significant changes to potential biomarkers, contributing to the therapeutic effects. Notably, QFDYDs more effectively normalized metabolic changes in response to lung injury and restored physiological function. The reliability of the model was confirmed by 200× permutation testing, which showed no overfitting (*R* ^2^ < 0.5 and *Q* ^2^ < 0.05) (Supplementary Figures S4A,B).

**FIGURE 7:**
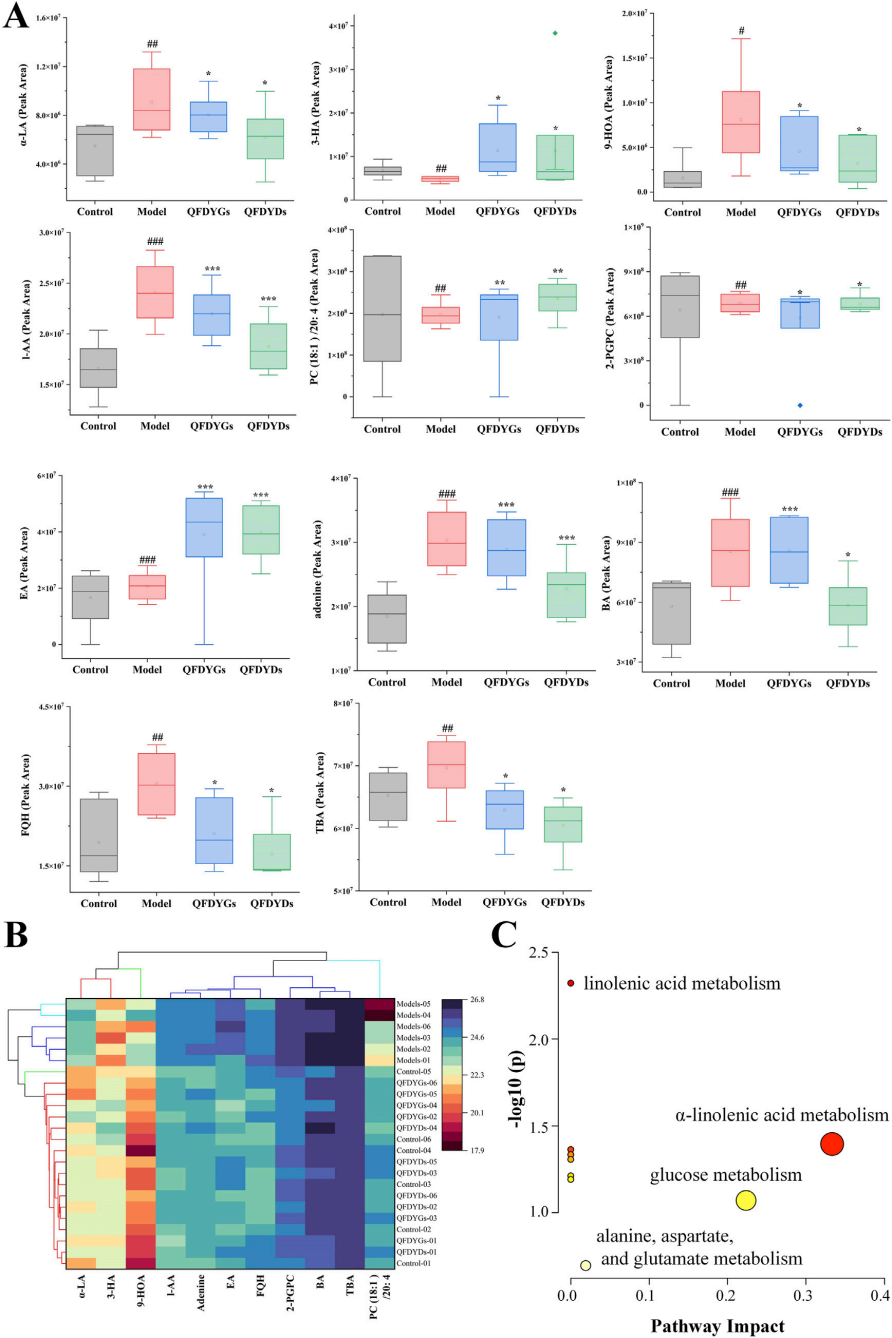
Analysis of 11 differential metabolites. **(A)** Box plots of the peak areas. **(B)** Cluster analysis. **(C)** Enrichment of metabolites. Data are presented as mean ± SD, n = 6. ^*#*^ *p* < 0.05, ^*#*^ *p* < 0.01, ^*###*^ *p* < 0.001 vs. control group; *p < 0.05, **p < 0.01, ***p < 0.001 vs. model group.

Based on OPLS-DA VIP > 1 and *t*-test *p* < 0.05 (Wu et al., 2022), 11 endogenous metabolites were identified as potential biometabolites of serum samples from mice with ALI after treatment with QFDYGs and QFDYDs. In the model group, the levels of α-linolenic acid (α-LA), 9-hydroxy-10,12-octadecadienoic acid (9-HOA), L-aspartic acid (L-AA), 2-palmito glycerophosphorylcholine (2-PGPC), eicosapentaenoic acid (EA), adenine, benzoic acid (BA), fumiquinazoline H, and tetrabromobisphenol A were markedly higher as compared to the control group (*p* < 0.05, *p* < 0.01, or *p* < 0.001), while 3-hydroxybutyric acid (3-HA) and PC (18:1 (11Z)/20:4 (5Z, 8Z, 11Z, 14Z)) (PC (18:1)/20: 4) were lower. Treatment with QFDYGs and QFDYDs restored the levels of these metabolites to varying extents (Figure 7A).

The data of 11 metabolite peak areas after normalization were used as variables for cluster analysis of 24 samples using Ward clustering and Pearson correlation with OriginPro 2024b software. The results showed that the six samples in the model group were clustered together, while the other 18 samples showed no obvious clustering pattern, indicating that after drug treatment, these metabolites were close to those in the control group. The 11 metabolites were divided into three groups with α-LA, 9-HOA, and 3-HA in one group, PC (18:1)/20:4 in a second group, and the remaining seven metabolites in third group (Figure 7B).

Metabolic pathway analysis of all endogenous metabolites was performed with MetaboAnalyst software (Gong et al., 2024). The metabolic pathways altered by ALI-induced pathological changes and modulation by treatment with QFDYGs and QFDYDs are illustrated in Figure 7C. The results indicate that α-LA metabolism and glucose metabolism are crucial pathways in ALI pathogenesis and treatment with QFDYGs and QFDYDs.

### 3.7 Correlation analysis between metabolomics and gut microbiota

**FIGURE 8:**
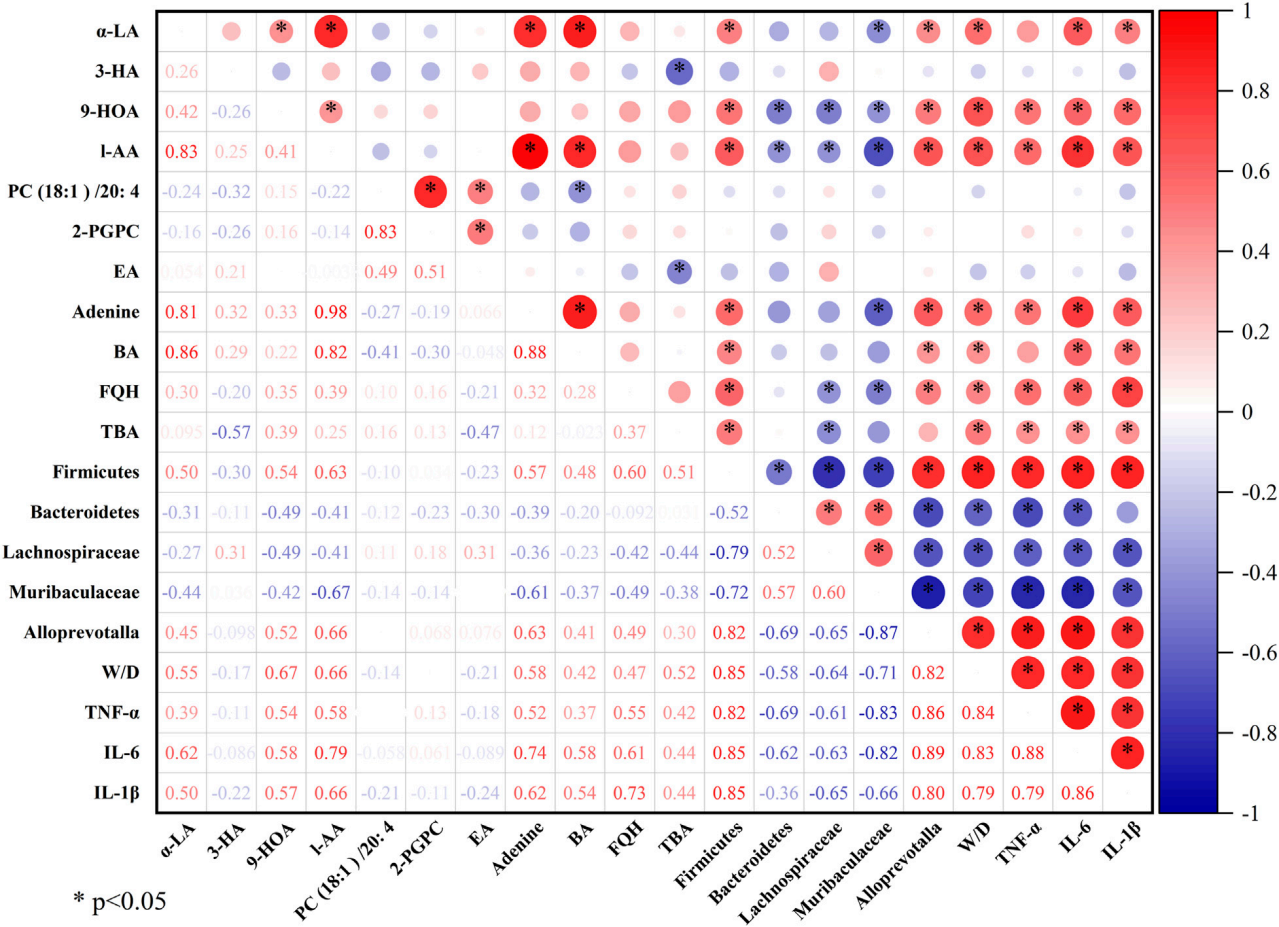
Correlation coefficients between metabolomics and gut microbiota regulated by QFDYGs.

Pearson correlation analysis was performed using OriginPro 2024b software to further explore the correlations among the metabolites and gut microbiota regulated by QFDYGs. As shown in Figure 8, among the 190 metabolite-bacteria pairs, 97 were positively correlated (*R* > 0) and 93 negatively correlated (*R* < 0), with 99 pairs being statistically significant (*p* < 0.05 or *p* < 0.01). The metabolites (except 3-HA and PC (18:1)/20:4) were generally positively correlated with *Firmicutes*, *Alloprevotalla*, the W/D ratio, TNF-α, IL-6, and IL-1β, and negatively with *Bacteroidetes*, *unclassified_Lachnospiraceae*, and *unclassified_Muribaculaceae*.

## 4 Discussion

ALI, a severe condition that can lead to life-threatening pulmonary disease or as a pulmonary manifestation of multiple organ dysfunction, is characterized by uncontrolled and self-amplifying lung inflammation, contributing to high morbidity and mortality among critically ill patients and posing a significant public health burden (Ma et al., 2023; Qin et al., 2024). Therefore, it is crucial to develop new drugs or therapeutic strategies against ALI. QFDYGs originates from the TCM theory of “epidemic-toxin obstructing lung.” The sovereign-minister combination simultaneously resolves inflammation, dissolves phlegm and protects the mucosal barrier, meeting the multi-target demands of TCM (Zhu et al., 2024). Here, we first demonstrate in the LPS-ALI model that both the granules (QFDYGs) and the decoction (QFDYDs) markedly lower lung-injury scores, curb neutrophil infiltration, suppress TNF-α/IL-6/IL-1β, and restore SOD and GSH, proving that core efficacy is independent of dosage form and readily translatable to the clinic.

The inflammatory response in ALI is largely mediated by the TLR4 signaling pathway. LPS, a potent inflammatory trigger, binds to TLR4 on host cell membranes, initiating a cascade of protein kinase reactions that ultimately activate the TLR4/NF-κB signaling pathway (Cai et al., 2024). TLR4 can activate both MyD88-dependent and TRIF-dependent pathways, with MyD88 playing a critical role in inflammatory signaling pathways (Xiong et al., 2022). In this study, LPS-induced ALI significantly upregulated expression of the TLR4, MyD88, and NLRP3 proteins, along with increased NF-κB phosphorylation, leading to an inflammatory storm characterized by excessive secretion of inflammatory factors, pulmonary epithelial cell damage, and increased vascular permeability. Both QFDYGs and QFDYDs effectively inhibited the TLR4-TRIF/MyD88-NF-κB-NLRP3 signaling pathway, thereby reducing inflammatory factor secretion and attenuating pulmonary edema. However, extra refining of QFDYGs raises levels of baicalin, baicalein, mangiferin, paeoniflorin, polydatin and timosaponin BII, giving it an edge in clearing oedema, repairing barriers and more potently down-regulating TLR4, MyD88 and p-NF-κB, thereby more completely blocking the TLR4-MyD88-NF-κB-NLRP3 axis at a lower dose. QFDYDs, rich in high-molecular polysaccharides and abundant hesperidin and procyanidin B1, acts rapidly and exerts robust anti-inflammatory effects.

Emerging evidence supports the gut-lung axis theory, which posits bidirectional communication between the gut and lungs in both health and disease (Budden et al., 2017; Qiu et al., 2022). The gut microbiota plays pivotal roles in immune system development and function, producing neurotransmitters and metabolites that influence neural, immune, endocrine, and metabolic pathways (Ma et al., 2022; Wang L. et al., 2023). Dysbiosis of the gut microbiota can exacerbate lung injury, as observed in this study, where LPS-induced ALI disrupted gut microbiota composition, reducing the abundance of beneficial bacteria (e.g., *Firmicutes*) and increasing harmful bacteria (e.g., *Bacteroidetes*) (Bahar-Tokman et al., 2022; Kapoor et al., 2023). Also, QFDYGs demonstrated superior efficacy in restoring the abundance of *Firmicutes* as compared to QFDYDs. Furthermore, both QFDYGs and QFDYDs restored gut microbiota balance, increasing the abundance of anti-inflammatory genera, such as *Blautia* and *Faecalibaculum*, which produce short-chain fatty acids with protective effects on intestinal barrier function and systemic immunity. These findings underscore the importance of gut microbiota modulation in ALI treatment and suggest that granule formulations may achieve comparable or superior effects as compared to decoctions.

Metabolomic analysis further elucidated the mechanisms underlying the therapeutic effects of QFDYGs and QFDYDs. ALI-induced dyslipidemia was evidenced by upregulated serum eicosapentaenoic acid levels and disrupted lysophospholipid metabolism. QFDYGs significantly elevated serum levels of 3-HA, a ketone molecule with anti-inflammatory properties (Jung et al., 2023), and normalized L-AA levels, which play a regulatory role in amino acid metabolism (Wang Y. et al., 2023). Additionally, QFDYGs alleviated disruptions to glycerophospholipid metabolism, as demonstrated by normalization of the serum levels of PC (18:1)/20:4 and 2-PGPC, which are closely associated with inflammatory responses and ALI pathogenesis (Hsu et al., 2024).

Pearson correlation analysis revealed significant and regular associations between metabolites and the intestinal flora, indicating that both formulations share a gut-lung-axis-metabolism network. QFDYDs preferentially drive micro-ecological and metabolic reprogramming and exhibit superior suppression of early-stage cytokines, suggesting they may be better suited to the initial inflammatory-storm phase. However, mechanistic confirmation will require quantifying downstream mediators of key metabolites in future work. By contrast, QFDYGs appear more beneficial for prolonged disease courses accompanied by barrier dysfunction. Taking together equivalent overall efficacy, complementary mechanisms, lower daily dose and stable quality, QFDYGs can replace QFDYDs, yet clinical choice should still be individualized according to TCM pattern differentiation.

## 5 Conclusion

**FIGURE 9:**
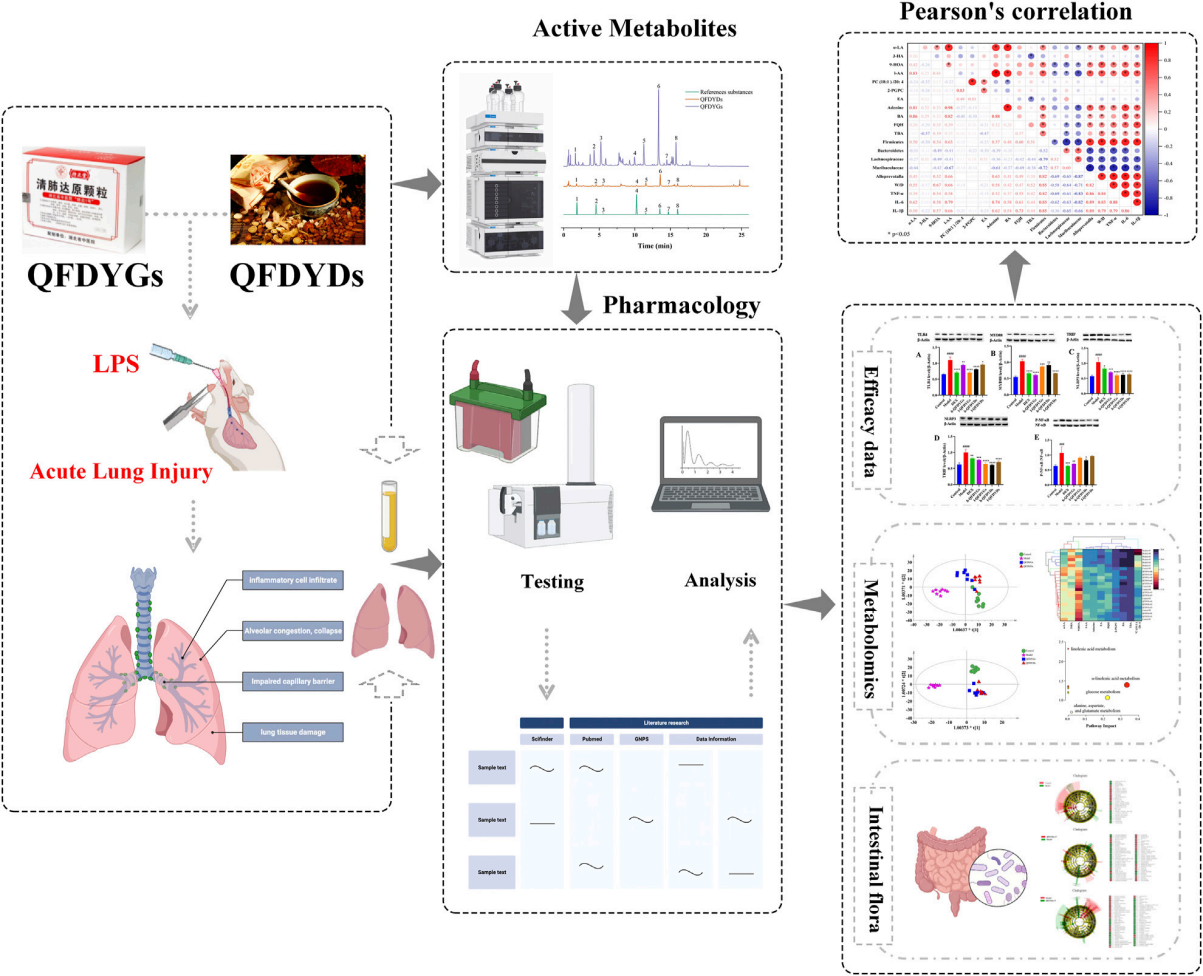
Workflow diagram for elucidation of the mechanisms underlying the therapeutic effects of QFDYGs and QFDYDs in ALI.

Our findings demonstrate that both QFDYGs and QFDYDs exert protective effects against ALI through modulation of the TLR4 signaling pathway, restoration of gut microbiota balance, and regulation of metabolic pathways. Notably, QFDYDs showed superior anti-inflammatory effects, demonstrating more effective targeting of cytokine-driven inflammation, whereas QFDYGs more effectively addressed oxidative stress and inhibition of the TLR4-TRIF/MyD88-NF-κB-NLRP3 signaling pathway. Both can potentially alleviate ALI-induced damage by interfering with the metabolic pathways, alongside improving intestinal microbiota composition and functionality (Figure 9).

The results indicate that granule formulations could be effective alternatives to traditional decoctions, with equivalent or superior therapeutic potential. More research is needed to confirm these results and assess the clinical use of granule formulations for ALI treatment. This study offers preliminary insights into the molecular mechanisms of QFDYGs in ALI treatment, supporting redevelopment and broadening potential applications. The choice between QFDYGs and QFDYDs should be guided by specific clinical presentations and therapeutic goals.

## Data Availability

The data presented in the study are deposited in the NGDC (BioProject) repository, accession number PRJCA050142. Further inquiries can be directed to the corresponding author.
