# Supplementary material for: Qingfei Dayuan granules and decoction alleviate acute lung injury via TLR4 signaling pathway modulation, gut microbiota regulation, and metabolic reprogramming
Source: Front Pharmacol. 2025 Nov 12;16:1643544. doi: 10.3389/fphar.2025.1643544 (PMC12647075; doi:10.3389/fphar.2025.1643544)
Supplement: Supplementary file 1 [file DataSheet1.docx]

Supplementary Material

**Supplementary Table 1.** Statistical analysis of alpha diversity index

| **Groups** | ACE | Chao1 | Simpson | Shannon | PD whole tree | Coverage |
| --- | --- | --- | --- | --- | --- | --- |
| Control1 | 416.3373 | 414.3333 | 0.9781 | 6.8157 | 25.9334 | 0.9998 |
| Control2 | 410.9466 | 403.8571 | 0.9812 | 6.8532 | 24.8428 | 0.9998 |
| Control3 | 390.4952 | 429.1714 | 0.9657 | 6.0416 | 27.6828 | 0.9999 |
| Control4 | 375.3471 | 429.5652 | 0.9674 | 6.1209 | 20.6761 | 0.9998 |
| Control5 | 439.9906 | 433.2500 | 0.9848 | 7.0857 | 23.5530 | 0.9997 |
| Control6 | 397.1913 | 391.6765 | 0.9849 | 7.0228 | 22.6960 | 0.9998 |
| Model1 | 368.9135 | 364.5385 | 0.9703 | 6.5399 | 34.5453 | 0.9998 |
| Model2 | 386.2946 | 383.3846 | 0.9845 | 6.9430 | 26.7572 | 0.9999 |
| Model3 | 325.6876 | 325.0208 | 0.9764 | 6.4477 | 35.1823 | 1.0000 |
| Model4 | 322.2951 | 319.1379 | 0.9829 | 7.0103 | 22.8086 | 0.9998 |
| Model5 | 289.7690 | 285.6071 | 0.9590 | 5.9246 | 21.5392 | 0.9999 |
| Model6 | 361.8589 | 360.2174 | 0.9759 | 6.5157 | 28.0123 | 0.9999 |
| QFDYGs1 | 368.0000 | 368.0000 | 0.9802 | 6.3550 | 20.9134 | 1.0000 |
| QFDYGs2 | 365.1784 | 362.3846 | 0.9767 | 6.4966 | 24.1332 | 0.9999 |
| QFDYGs3 | 387.3463 | 355.1176 | 0.9763 | 6.5581 | 23.3471 | 0.9999 |
| QFDYGs4 | 393.4212 | 391.6667 | 0.9728 | 6.5343 | 25.3675 | 0.9998 |
| QFDYGs5 | 454.6667 | 473.5000 | 0.9883 | 7.3514 | 26.5783 | 0.9998 |
| QFDYGs6 | 399.8065 | 388.2353 | 0.9742 | 6.3422 | 21.3898 | 0.9998 |
| QFDYDs1 | 458.7551 | 455.3000 | 0.9904 | 7.5301 | 25.4726 | 0.9998 |
| QFDYDs2 | 373.6371 | 371.7500 | 0.9819 | 6.7178 | 24.5805 | 0.9999 |
| QFDYDs3 | 379.0000 | 379.0000 | 0.9633 | 6.0833 | 25.7216 | 1.0000 |
| QFDYDs4 | 345.9474 | 345.2143 | 0.9786 | 6.6435 | 35.8963 | 1.0000 |
| QFDYDs5 | 338.3152 | 378.0000 | 0.9751 | 6.2603 | 26.7656 | 1.0000 |
| QFDYDs6 | 523.7321 | 443.0152 | 0.9859 | 7.1875 | 45.6186 | 1.0000 |


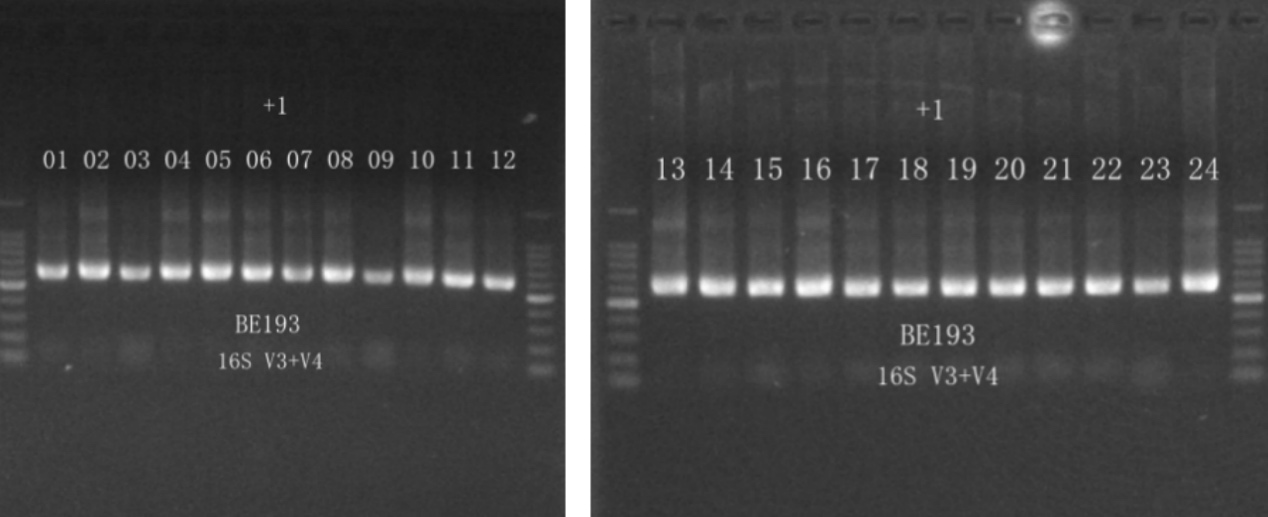


**Supplementary Figure 1.** Total DNA electrophoresis of samples.


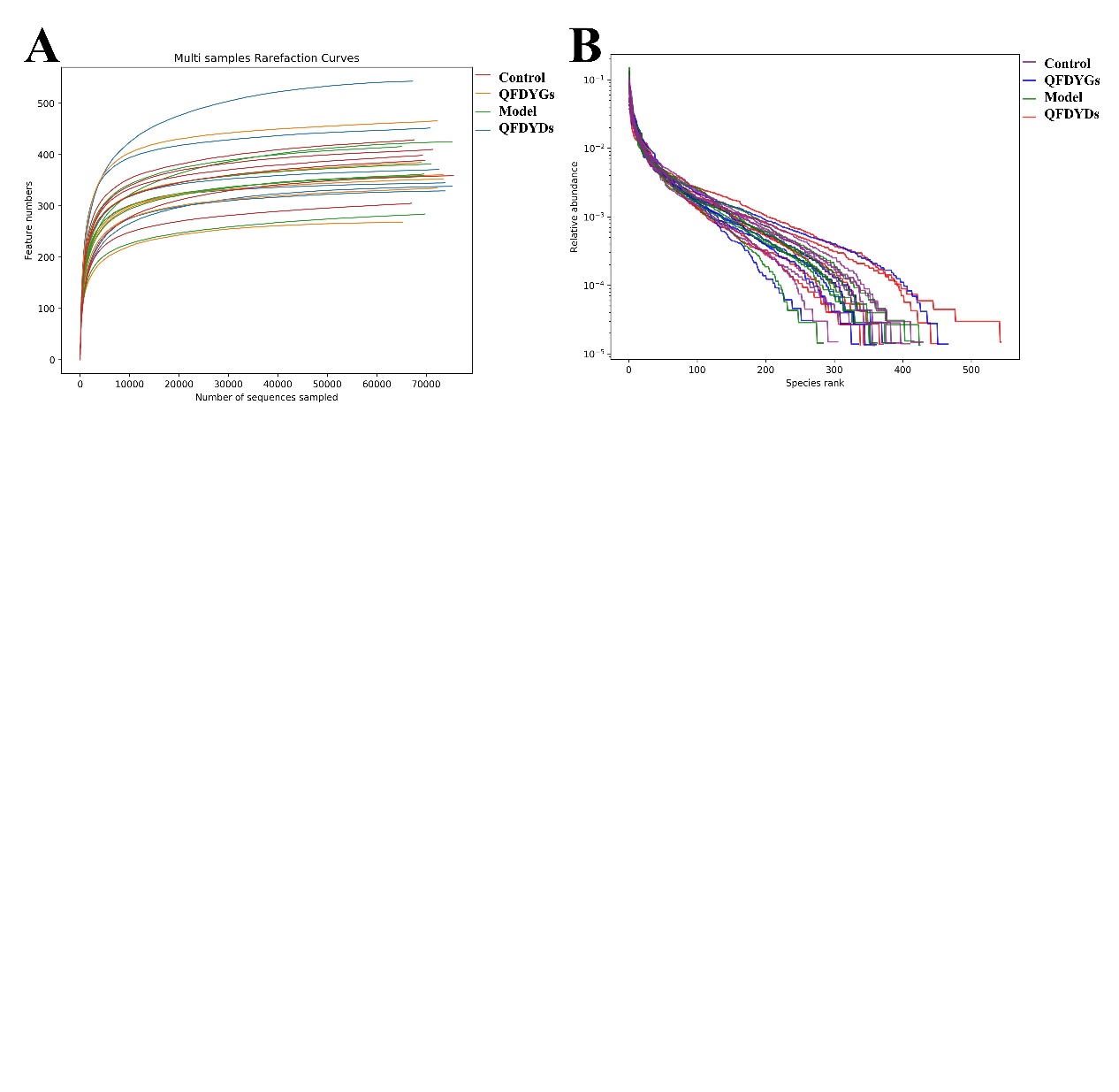


**Supplementary Figure 2.** The diversity analysis of gut microbiota analysis. (A) Dilution curves. (B) Rank-abundance curves.


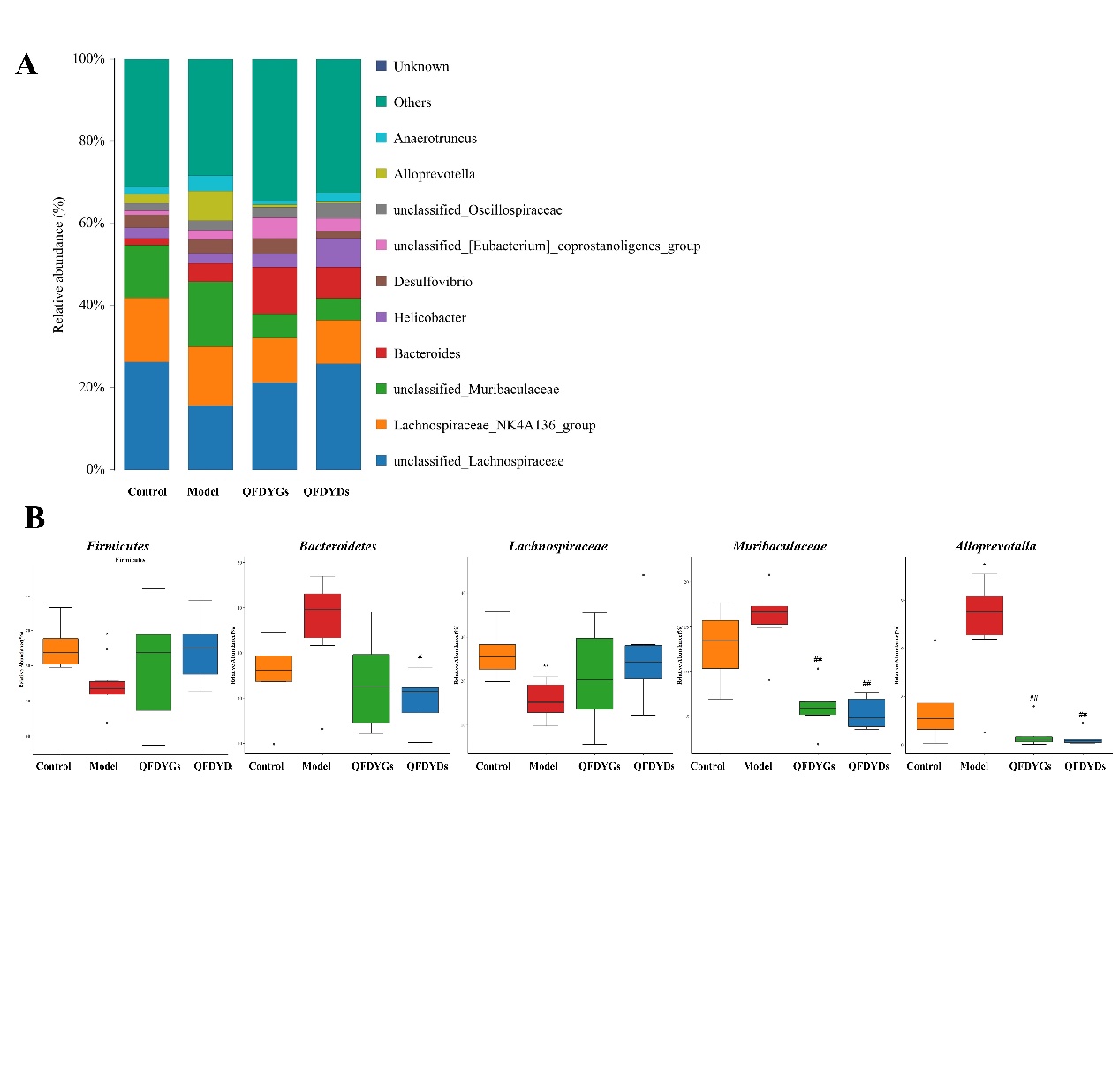


**Supplementary Figure 3.** (A) Relative abundances and composition of microbial communities at the phylum level. (B) Comparison of the abundance of intestinal flora differing at the phylum level and genus level. *^*^p* < 0.05, *^**^p* < 0.01 compared with group Control. *^#^p* < 0.05, *^##^p* < 0.01 compared with group Model.


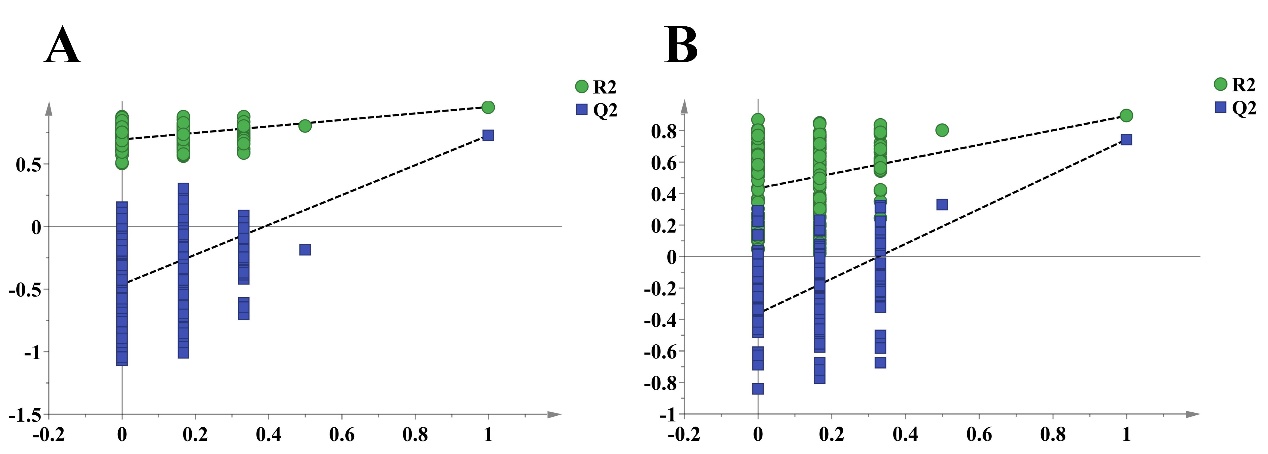


**Supplementary Figure 4.** Permutation testing. (A) positive ion mode. (B) negative ion mode.
